# Reliability Assessment of Wearable Technologies for Physiological Measurements: An Evaluation of Shimmer3 GSR+, Empatica E4, EmbracePlus, and Pixel Watch 2 Across Cognitive, Affective and Physical Activity Tasks

**DOI:** 10.3390/s26144376

**Published:** 2026-07-10

**Authors:** Charlotte Brandebusemeyer, Fabian Georgi, Bert Arnrich

**Affiliations:** Digital Health—Connected Healthcare, Hasso Plattner Institute, University of Potsdam, 14482 Potsdam, Germany; fabian.georgi@hpi.uni-potsdam.de (F.G.); bert.arnrich@hpi.de (B.A.)

**Keywords:** wearables, wristband, physiological data, PPG, EDA, skin temperature, empirical study

## Abstract

Wearable sensing has evolved from custom-built systems towards standardized research- and consumer-grade devices, improving accessibility and comparability in physiological data analysis. Wrist-worn sensors offer non-invasiveness, minimal setup, and suitability for everyday use, making them increasingly attractive for both research and real-world applications. However, it remains unclear how consumer-grade smartwatches compare with established research-grade wearables in physiological data quality and suitability for empirical studies. We present a mixed-methods study assessing the signal comparability and reliability of the research-grade Shimmer3 GSR+, Empatica E4, and the consumer-grade Pixel Watch 2 under controlled conditions including baseline, cognitive load (n-back), startle, relaxation and physical activity phases. A second study replicated key conditions to exploratorily compare the Empatica E4 with its successor, EmbracePlus. All wearables reliably captured pronounced physiological changes between distinct experimental conditions, but were less sensitive to subtle cognitive load differences and varied in absolute values and motion robustness. The Pixel Watch 2 effectively captured heart rate and skin temperature trends in dynamic settings, whereas research-grade devices remained preferable for analyses requiring raw data access, transparent data processing and advanced physiological metrics. Electrodermal activity provided the most consistent task differentiation under motion. Wrist-worn devices were perceived as more comfortable and less obtrusive than finger-based sensors.

## 1. Introduction

Wearable technologies have become increasingly popular, especially in the area of health and fitness monitoring. Equipped with small, precise sensors, these devices provide a non-invasive way to track movement, collect biometric data, and analyze daily activities. Among wearables, wristbands have emerged as the preferred choice due to their compatibility with everyday use, long battery life, and seamless wireless connectivity [1,2,3,4].

In research contexts, wearable sensing has historically relied on custom-built systems requiring substantial technical expertise for development, calibration, and data acquisition. While these systems provided full access to raw physiological signals and high flexibility, they were difficult to scale and reproduce. The introduction of standardized research-grade devices, such as the Shimmer3 GSR+ and Empatica E4, improved accessibility and comparability across studies. These devices enable high-precision physiological measurements and are widely used in the literature. For example, the Shimmer3 GSR+ has been extensively employed to measure physiological responses [5,6,7,8,9] and is an established research-grade wearable that has been widely used in laboratory and controlled studies involving photoplethysmography (PPG) and electrodermal activity (EDA) measurements [10,11,12]. Similarly, the Empatica E4 has established itself as a widely utilized and reliable research wearable, particularly for detecting stress [13,14,15,16,17] and various emotional states [18,19]. However, despite these advantages, research-grade devices remain expensive and require considerable expertise to deploy in study setups and to process the raw data, limiting their scalability and everyday use.

In recent years, the consumer market for wearable devices has rapidly evolved, with advancements leading to increasingly sophisticated sensor capabilities. Devices such as the Fitbit Sense, Fitbit Sense 2, and the Google Pixel Watch 2 exemplify this progress. These smartwatches are equipped with sensors measuring physiological parameters such as PPG and EDA, approaching the physiological measurement capabilities of research-grade devices. Notably, these consumer devices are significantly more affordable and scalable. However, they often provide limited access to raw data and rely on proprietary processing pipelines, raising questions about their suitability for scientific use.

Despite these advances, there is a lack of systematic evaluation and comparison of research and consumer wearables in realistic everyday scenarios. In particular, the reliability of measuring cognitive load and startle responses using physiological signals such as EDA, PPG, and skin temperature (TEMP) remains underexamined. Furthermore, while the Empatica E4 has been widely used in research, it has become outdated, and it remains unclear how its successor, the EmbracePlus, compares to established research-grade devices.

To address these gaps, we conducted a controlled study comparing the Shimmer3 GSR+, Empatica E4 and Google Pixel Watch 2 across simulated everyday tasks, including a baseline condition (watching a nature video), three levels of n-back tasks to induce cognitive load, a startle event, a breathing-based relaxation phase, and a physical activity condition. We analyzed differences in physiological responses across devices and study conditions. To compare the outdated Empatica E4 wristband with the new EmbracePlus wristband, we conducted a second study, using the same study setup for the three cognitive load n-back tasks, the startle event, and the breathing relaxation phase. Behavioural and subjective measures were additionally collected and analyzed to validate that the experimental tasks elicited the intended cognitive load and startle responses, thereby providing context for interpreting the physiological device comparisons. Additionally, all four devices were evaluated in terms of perceived comfort.

The following research questions are going to be addressed:

**(RQ1)**  Do the behavioural and subjective responses demonstrate that the study design is effective for eliciting task-related physiological responses?
**(RQ2)**  How comparable and reproducible are physiological measurements across research-grade and consumer wearables in capturing task- and event-related physiological responses, and where do devices show limitations?
**(RQ3)**  To what extent does the EmbracePlus reproduce physiological measurements from the outdated Empatica E4 during cognitive n-back tasks, a startle event, and a relaxation phase?
**(RQ4)**  How do participants perceive the comfort of the wearables?

This empirical study aims to investigate the reliability of research- and consumer-grade wearables for measuring physiological differences during everyday tasks. Specifically, we see our contribution in

- Systematic evaluations of the wearable devices Shimmer3 GSR+, Empatica E4, EmbracePlus and Google Pixel Watch 2 with regard to their physiological signal quality and agreement in reflecting expected physiological changes during simulated everyday tasks.
- Assessment of the ability of the devices to capture extreme scenarios (startle event) and subtle cognitive load differences (n-back tasks).
- Comparison of the outdated Empatica E4 wristband with its successor EmbracePlus to evaluate the reliability across device generations.
- A multimodal approach with a focus on physiological data analysis that is strengthened with behavioural and subjective data.

## 2. Related Work

Several validation studies have evaluated the performance of research-grade devices. Milstein and Gordon [20] assessed the Empatica E4 wristband as a tool for measuring cardiac interbeat intervals (IBIs), heart rate variability (HRV), and EDA, comparing it to the well-validated MindWare mobile impedance cardiograph. Data from 30 participants were collected during rest and social conversations to evaluate the E4’s performance in more natural, interactive settings. The study found that IBI and HRV measurements from the E4 showed moderate to high agreement with the reference device, particularly during rest. However, the EDA data from the E4 were unreliable, suggesting limitations in capturing skin conductance during interactions involving hand movements.

Sanchez-Comas et al. [21] compared the Shimmer3 GSR+ and Empatica E4 in measuring EDA, collecting data from 17 participants exposed to both pleasant and unpleasant stimuli. Each participant wore four EDA sensors simultaneously, with measurements taken from different body sites. The study found that the Shimmer3 GSR+ provided more reliable and consistent signals, while the Empatica E4 showed moderate correlations and occasional anomalies, potentially due to its lower sampling rate.

Giorgi et al. [12] evaluated the reliability of the Empatica E4 and Shimmer device, alongside other consumer and research-grade wearables, in assessing mental and emotional states. Data from 17 participants were collected during simulated work scenarios designed to induce varying levels of stress, workload, and emotional states. The study focused on collecting electrooculographic (EOG), EDA, and PPG signals. The results demonstrated a statistically significant correlation between the Empatica E4 and the Shimmer3 GSR+.

Ronca et al. [10] laid the foundation for this work by comparing research-grade devices (Empatica E4 and Shimmer3 GSR+) to a consumer-grade wearable, the Fitbit Sense. However, the study has limitations: first, participants were instructed not to perform any activities during the recording, creating an artificial setting that limits the generalizability of the results to real-life scenarios. The data collection consisted of only three 90 s recordings per participant. As measurements were estimated at 30 s intervals, this resulted in just nine data points per individual. This severely limited the dataset size and reduced the ability to conduct a thorough evaluation of the wearables’ performance.

A recent study by Sinichi et al. [22] tested four PPG-based heart rate wearables (three consumer devices: Kyto2935, Schone Rhythm 24, HeartMath Inner Balance Bluetooth; one research-grade: Empatica EmbracePlus) on 40 participants and compared them against an electrocardiogram (ECG) device (VU-AMS). Participants underwent rest, walking, cognitive and ambulatory-like tasks to assess heart rate (HR) and HRV measurements. They found that the EmbracePlus did not outperform the consumer-grade devices and that agreement with ECG was limited, especially under movement. This study does not consider further modalities beyond heart-activity-related measures.

Prior work has not systematically compared research- and consumer-grade wearables across multiple physiological signals—including heart rate, electrodermal activity, and skin temperature—during simulated everyday tasks. In particular, there is limited evidence on the reliable measurement of startle events and cognitive load using n-back tasks. Furthermore, no study has directly compared the widely used, now outdated Empatica E4 with its successor, the EmbracePlus, under comparable conditions. These gaps motivate the need for a comprehensive evaluation of both research- and consumer-grade devices in ecologically valid task scenarios.

## 3. Materials and Methods

### 3.1. Research Design

The first empirical study (study 1) aims to test the Shimmer3 GSR+ (Shimmer Sensing, Dublin, Ireland), the Empatica E4 (Empatica Inc., Boston, MA, USA), and the Google Pixel Watch 2 (Google LLC, Mountain View, CA, USA) in diverse situations to uncover potential differences in their performance across varying conditions. To ensure the relevance of the study to everyday life, participants were exposed to five scenarios that commonly occur in real-life settings: resting (baseline measurement), cognitive load, startle event, relaxation, and physical activity. During all scenarios, participants simultaneously wore the three devices. This setup enabled a direct comparison of the measurement agreement of the devices in each scenario.

The second empirical study (study 2) had a different main research aim but included the same cognitive load, startle event, and relaxation study setup as in the first study. A different participant cohort from the first study wore the EmbracePlus wristband (Empatica Inc., Boston, MA, USA). The aim of including parts of the first study in the second was to validate and compare the quality of the EmbracePlus wristband with that of its predecessor, the Empatica E4, and the other devices.

Both studies, including study design, study procedure and data protection considerations, have been approved by the ethics committee of the University of Potsdam.

### 3.2. Experimental Setup

Participants in the first study engaged in five different situations, each selected to examine different aspects of physiological responses. Three of these situations were also considered in the second study. These situations cover a range of activities, enabling a detailed comparison of the measurements from each device under various conditions. Questionnaires were administered at various stages of the experiment to capture participants’ subjective experiences, and behavioural data were gathered during the cognitive load n-back tasks. A web application was developed to ensure a consistent setup throughout the study, automatically displaying the questionnaires at the appropriate times, reducing human error and ensuring smooth progression through the experiment.

#### 3.2.1. Baseline Rest

The baseline phase at the beginning of the first study enabled participants to disengage from daily activities and acclimatize to the experimental setting, while capturing physiological signals under a neutral condition. Participants were seated at a laptop and were instructed to sit comfortably, refrain from speaking or moving, relax and breathe spontaneously. To reduce mind-wandering and disruptive thoughts, a five-minute video of natural landscapes was shown, providing a calming yet engaging stimulus. Prior studies have demonstrated that exposure to nature (either directly or through images) can reduce physiological stress [23,24,25], supporting this design choice for the baseline phase.

During the second study, acclimatization of the participants to the experimental setting was achieved via having them fill in a longer pre-questionnaire followed by a shorter 2 min natural landscape video.

#### 3.2.2. Cognitive Load n-Back Tasks

The n-back task is a widely used paradigm for assessing cognitive load and working memory. Originally introduced by Kirchner [26], it requires participants to monitor a sequence of stimuli (e.g., letters) and indicate whether the current stimulus matches the one presented *n* items earlier. Task difficulty increases with *n*, as higher levels require continuous updating and maintenance of information in working memory. Due to its controllable difficulty, the n-back task is widely used to induce and measure cognitive load [27,28,29,30].

Our implementation of the n-back task is based on [29], but was adapted to increase the difficulty. The three difficulty levels (1-back, 2-back and 3-back) were presented in 2 min blocks. In each block, 48 letters were shown, 16 of which were target stimuli, meaning they matched the stimulus presented *n* positions earlier. Each letter was displayed for 0.5 s, followed by a 2 s inter-stimulus interval during which a fixation cross appeared. During the 2.5 s, participants were supposed to indicate as quickly as possible if the shown letter matched the one *n* letters earlier. Pressing the right arrow on the keyboard indicated a perceived target, and pressing the left arrow indicated a non-target letter. The number of errors was displayed to the participants to keep them motivated.

For each difficulty level, we recorded response time, response availability within the allowed interval, and response accuracy. After each n-back task, participants completed a questionnaire assessing perceived cognitive load and frustration on a 7-point Likert scale (1 = very low, 7 = very high). These measures enabled comparison of objective behavioural performance (accuracy and response time) with subjectively perceived workload across difficulty levels. Furthermore, the behavioural and subjective data were used to analyze whether the study design was effective so that physiological responses could be expected.

#### 3.2.3. Startle Event

The startle event was included to elicit a strong physiological response and evaluate the devices’ ability to capture rapid signal changes. An acoustic honk sound was used as a natural stimulus that can occur in an urban environment. The sound was played directly after the 3-back task. Participants were not informed about a startle event prior to the start of the study to produce an authentic reaction. After the honk, participants were immediately informed that the sound was intended to measure their physiological responses. They were then asked whether they heard the honk (yes/no) and, if so, how startled they were (7-point Likert scale: 1 = not at all, 7 = extremely).

#### 3.2.4. Breathing Relaxation

After the n-back tasks and the startle event, we included a 5 min relaxation phase to broaden the range of recorded conditions and allow physiological signals to return to a relaxed level. Including a recovery period after a reactive phase facilitates clearer differentiation between conditions [31]. Participants practiced a video-guided 4-7-8 breathing technique (inhale 4 s, hold 7 s, exhale 8 s) corresponding to slow, deep breathing at approximately three beats per minute. This breathing pattern has been shown to reduce physiological stress, including decreases in heart rate and blood pressure [32,33,34,35].

#### 3.2.5. Physical Activity

The 5 min physical activity provided a non-stationary condition in the first study. To minimize motion artefacts in the physiological signals, particularly due to excessive arm movements, participants were instructed to jog in place with their hands resting on the back of a chair. To make the task more engaging, participants watched a point-of-view video of a runner moving through a scenic mountain landscape, accompanied by energetic music.

#### 3.2.6. Web Application

To ensure a consistent procedure across study conditions, we developed a web application to guide participants through the study and standardize the experimental protocol. The application was implemented using Next.js and Tailwind and deployed online via Vercel.

The interface follows a minimal, single-page design with a neutral background to reduce distractions from the tasks. Before each activity, participants received brief on-screen explanations and instructions, eliminating the need for experimenter interaction. This reduces potential experimenter-induced bias and ensures that changes in physiological data can be attributed to the activity rather than external factors.

The system automatically generated Unix timestamps at the start of each activity, enabling precise synchronization with physiological data. This sub-second precision is particularly important for short-lived events such as startle responses and eliminates manual timing errors.

The application also supported automated study management. Each participant was assigned a random identifier to ensure anonymity. To reduce systematic bias, devices were randomly assigned to participants’ arms: the Pixel Watch 2 and Empatica E4 were placed on opposite arms with randomized assignment, while the Shimmer3 GSR+ was worn on the non-dominant hand (or randomly assigned if no dominance was reported). Questionnaire and behavioural data were collected digitally, reducing errors such as illegible handwriting.

At the end of each session, all data were automatically exported to a CSV file, including participant ID, device assignments, activity start times, questionnaire responses, and behavioural data. This automated export ensures consistent data collection and reduces errors from manual handling. To protect privacy, all data were stored locally and not transmitted to any external database or service.

The web application was slightly adapted for the second study to fit into the study procedure: Participants entered their personal study identifier, and the baseline and physical activity conditions were removed. The remaining functionalities remained unchanged from the first study.

### 3.3. Participants

#### 3.3.1. Cohort for Study 1

Twenty participants (7 female, 13 male) aged 18–33 years (M = 23.75) took part in the study and wore the Shimmer3 GSR+, Empatica E4 and the Google Pixel Watch2 throughout the study. Regarding handedness, 17 participants were right-handed, two were left-handed, and one reported mixed-handedness. Most participants were students at the Hasso Plattner Institute in Potsdam, specializing in computer science, digital health or related fields.

To be eligible for the study, participants had to meet several inclusion criteria: They needed to be at least 18 years old, in good general health, fluent in English at a B2 level or higher, and physically capable of jogging for five minutes at a moderate pace. Participants with a history of mental disorders, neurological diseases, or cardiovascular conditions or potential dependency on the experimenter were excluded.

Participants were recruited through advertisements distributed via mailing lists and personal contacts. In appreciation of their participation, each participant received a 30-euro Amazon voucher.

#### 3.3.2. Cohort for Study 2

Twenty-two professional software developers from SAP constructed the second participant cohort, who solely wore the EmbracePlus wristband. The participants were part of another extensive study [36], which consisted of a section that replicated the cognitive n-back tasks, the startle event and the relaxation breathing phase of the first study. In the study setup and procedure, we refrained from collecting age and gender information for data privacy reasons. Participants also received an Amazon voucher for their endeavours at the end of the study.

Four participants needed to be excluded from the evaluations in this paper: one participant did not react to over 1/3 of the n-back task letters, and two participants were excluded as outliers due to extremely high error rates across all three n-back tasks. All three participants thereby indicated difficulty in understanding the n-back task study setup. For one participant, the physiological signals were too distorted during the tasks. Eighteen participants remained in the second cohort for analyses.

### 3.4. Devices

#### 3.4.1. Shimmer3 GSR+

**Figure 1:**
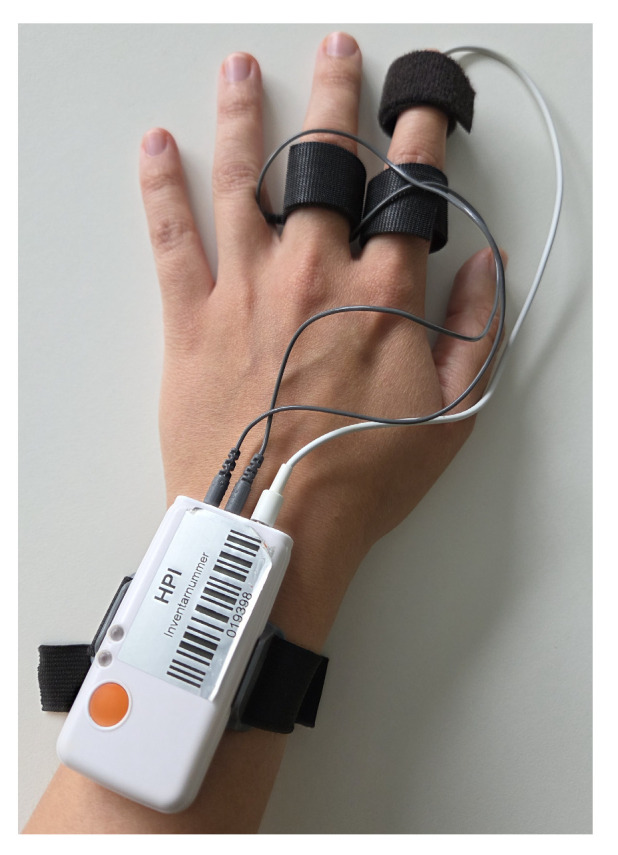
Shimmer3 GSR+ device.

Shimmer3 GSR+ (Shimmer Sensing, Ireland) is a wrist-worn wearable capable of recording PPG, EDA, 3-axis acceleration and internal temperature (Figure 1). Following the procedure by Udovičić et al. [6] PPG is measured at the palmar side of the index finger and EDA at the palmar surface of the index and middle fingers. Data were collected at 252 Hz in streaming mode via Consensys software v1.6.0 [37] and exported as CSV for custom analysis. Other sensors (e.g., magnetometer, angular velocity, pressure) were not used in this study. This wearable device is an established research-grade device frequently used in laboratory studies for PPG and EDA measurements [10,11,12].

#### 3.4.2. Empatica E4 and EmbracePlus

**Figure 2:**
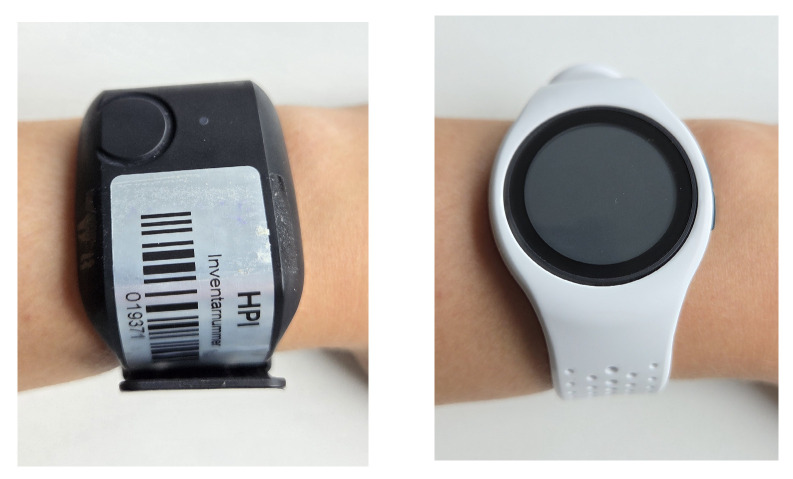
Empatica E4 wristband (**left**) and the EmbracePlus wristband (**right**).

The Empatica E4 wearable wristband (Figure 2, left) is equipped with sensors to measure PPG at 64 Hz, skin temperature at 4 Hz and 3-axis acceleration at 32 Hz on the outside of the wrist and EDA on the inside of the wrist at 4 Hz. The raw data is assessable in CSV format. The device has established itself as a widely utilized tool in research and has demonstrated reliability in the detection of stress [13,14,16,17] and various emotional states [18,19,38]. The device has, however, been discontinued, with support ending in February 2025. The succeeding device is the EmbracePlus wristband.

The EmbracePlus wristband (Figure 2, right) records PPG data at 64 Hz, EDA at 4 Hz, skin temperature at 1 Hz and 3-axis acceleration at 64 Hz at the same locations on the wrist. Raw data from each sensor is stored in an AVRO file format, with each file containing up to 30 min of continuous data before a new file is generated.

#### 3.4.3. Google Pixel Watch 2

**Figure 3:**
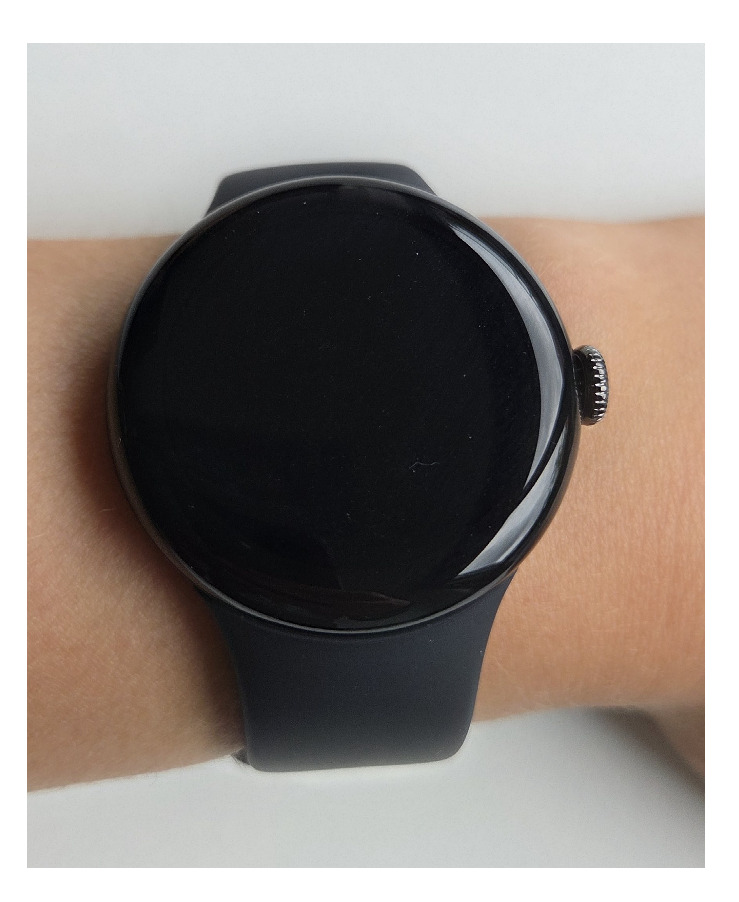
Google Pixel Watch 2 smartwatch.

The wrist-worn consumer-grade smartwatch Google Pixel Watch 2 (Google, released October 2023) (Figure 3) measures PPG, EDA, skin temperature and 3-axis acceleration. It was chosen because it was reported to be one of the few smartwatches equipped with an EDA sensor. Sensor data is recorded not at fixed sampling rates, but asynchronously and dynamically when new data becomes available. Raw data access is not natively provided, so we developed a custom Android application using the SensorManager API in Android [39] to collect asynchronous sensor events. Relative timestamps were converted to Unix timestamps to ensure temporal alignment with the other devices. The data were streamed to a connected smartphone to reduce I/O and conserve battery life of the watch, buffered and written to CSV files every six seconds. Most PPG and EDA data remain uninterpretable because Google and Fitbit do not disclose the sensor output format, so only heart rate, skin temperature, and accelerometer data could be considered for this study.

### 3.5. Measures

#### 3.5.1. Physiological Data

Physiological measures were calculated over 122.5 s time windows for task comparisons (corresponding to the length of one n-back task) and over 30 s windows for the startle event analysis. The chosen time windows provide a compromise, offering sufficient data for robust feature extraction while maintaining sensitivity to task-related changes. To account for skewness and improve statistical robustness, some of the following measures were log-transformed (details in Section 4.3.2 Linear Mixed-Effects Models). Table 1 gives an overview of the physiological parameters used in the analyses for each device.

##### Photoplethysmography (PPG)

PPG is an optical technique used to measure the blood volume pulse (BVP). Variations in light absorption enable the extraction of interbeat intervals (IBIs), i.e., the time between successive heartbeats. From IBIs, heart rate (HR) and heart rate variability (HRV) features were derived:

- **HR:** Mean heart rate;
- **RMSSD:** Root mean square of successive differences between normal-to-normal (NN) intervals;
- **pNN50:** Percentage of successive NN heartbeats that differ by more than 50 milliseconds.

##### Electrodermal Activity (EDA)

EDA reflects changes in the skin’s electrical conductance associated with sympathetic nervous system activity. The signal comprises a tonic component, the skin conductance level (SCL), and a phasic component, the skin conductance response (SCR). SCL captures slow baseline changes over time, while SCR represents rapid, event-related changes. The following metrics were analyzed:

- **SCL:** Tonic skin conductance;
- **CleanEDA:** Cleaned EDA signal (tonic and phasic component combined);
- **CountSCR:** Number of detected SCR peaks.

##### Skin Temperature (TEMP)

Skin temperature is influenced by environmental conditions and physical activity and varies in response to acute stress. As such, it is a useful indicator for assessing stress and emotional states. The following metrics were analyzed:

- **MeanTEMP:** Mean TEMP;
- **RangeTEMP:** Range of TEMP values.

##### Acceleration

Acceleration data was utilized to identify and remove motion artefacts in the other physiological signals that occur due to arm and wrist movements. Details on the processing pipeline can be found in Section 4 Data Processing.

#### 3.5.2. Behavioural Data

During the n-back tasks, we recorded response time and correctness. Response time was defined as the latency between stimulus onset and participant response. Correctness was categorized into three classes: correct response, incorrect response, and missed response (no answer within the allowed time window).

#### 3.5.3. Subjective Data

##### Pre-Questionnaire

In the first study, demographic information, including age, gender, and profession or field of study, was collected. Handedness was recorded to determine device placement. In the second study, participants’ professions and handedness were collected.

##### Cognitive Load and Startle Questionnaire

After each n-back task, participants responded to two questions. The first question assessed perceived cognitive load (“How would you rate your cognitive load during this set of tasks?”), adapted from [40]. The second question assessed frustration (“How insecure, discouraged, irritated, stressed, and annoyed were you?”), derived from the NASA Task Load Index (NASA-TLX) [41]. Both questions were rated on a 7-point Likert scale (1 = very low, 7 = very high). After the startle event at the end of the 3-back task, participants additionally answered two questions regarding the event. They first answered whether they perceived the honk sound (yes/no). If they did, they rated how much the sound startled them on a 7-point Likert scale (1 = not at all, 7 = extremely).

##### Post-Questionnaire

At the end of the first study, participants rated the comfort of the three devices on a 5-point Likert scale (1 = very uncomfortable, 5 = very comfortable). Additionally, participants provided qualitative feedback via three open-ended questions covering general impressions of the study, whether any unusual events occurred, and any additional comments or observations. In the second study, participants were asked in an open-ended question whether the wristband disturbed their work routine.

### 3.6. Study Procedure

**Figure 4:**
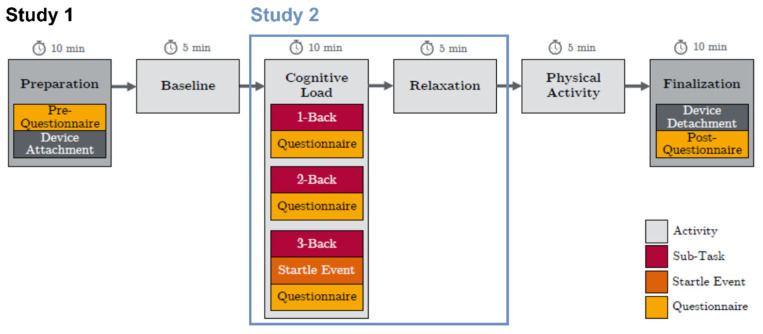
Overview of the study procedure. The procedure for study 1 is fully displayed, whilst the overlapping part of study 2 with study 1 is surrounded in blue.

The study procedure for study 1 is fully detailed below. The relevant, overlapping part of study 2 with study 1 is also described. An overview of both study procedures is shown in Figure 4.

#### 3.6.1. Study 1

The study was conducted at the Hasso Plattner Institute and lasted approximately one hour per participant. The study room was prepared according to best practices for electrodermal activity measurements [42], with the room temperature maintained at 23 °C. Environmental conditions were standardized by controlling lighting, minimizing external noise (e.g., closed windows, silence notices on the outside of doors), and ensuring all devices were fully charged and operational.

Upon arrival, participants were welcomed, given time to settle in and then seated. The experimenter provided an overview of the study procedure (excluding the startle event), after which participants gave informed consent to participate. To minimize study and signal interference, participants silenced or disabled personal devices.

**Figure 5:**
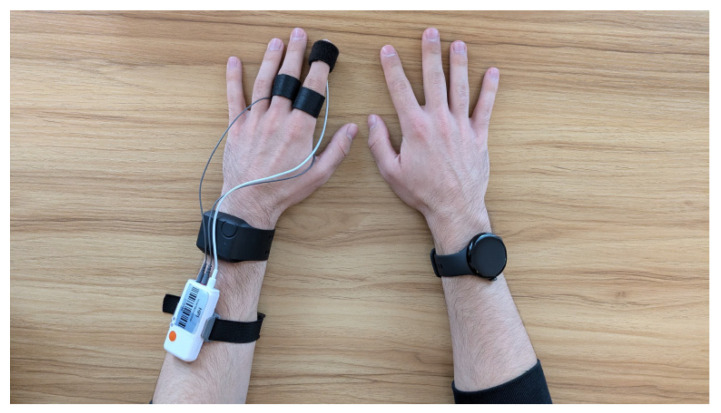
Example of a possible device attachment setup: Google Pixel Watch 2 on the right arm, Empatica E4 on the left arm and the Shimmer3 GSR+ worn above the Empatica E4 with the sensors attached to the fingers.

Participants then used the web application (see details in Section 3.2.6 Web Application) to guide them through the study. After completing a pre-questionnaire, wearable devices were attached (Figure 4). Device placement was partially randomized to reduce bias: the Pixel Watch 2 and Empatica E4 were worn on opposite arms with randomized assignment to which arm, while the Shimmer device was placed on the non-dominant hand (or randomly if no dominance was reported). An example of a possible device attachment setup is shown in Figure 5. Participants received final instructions, including to minimize movement and avoid moving or interacting with the sensors and devices. The experimenter remained in the room in case of any emergencies but did not interact with the participant during the tasks.

The study procedure (Figure 4) then consisted of a baseline phase, followed by three cognitive load-inducing n-back tasks of increasing difficulty. After each n-back task, the participants rated their perceived cognitive load and frustration. Following the 3-back task, a brief acoustic honk stimulus was presented to elicit a startle response. Participants subsequently rated the intensity of the startle event, then followed a guided breathing relaxation phase and a physical activity phase involving jogging in place.

At the end of the study, recordings were stopped, devices were removed, and participants completed a post-questionnaire. Participants received compensation and were then dismissed.

#### 3.6.2. Study 2

The study was conducted at SAP, with the part relevant to this paper spanning ~20 min (please find the complete study procedure in this paper [36]). Participants were seated in a room with stable environmental conditions. Physiological data were continuously recorded using the EmbracePlus wristband on the participant’s non-dominant wrist. With guidance from the web application, the same procedure regarding the n-back tasks, the startle event and the relaxation breathing phase as in study 1 was followed (Figure 4, blue surrounding).

## 4. Data Processing

Each physiological signal collected from the devices underwent a series of preprocessing steps to remove motion artefacts and implausible values. The cleaned signals were subsequently processed to derive the measures described in Section 3.5 Measures.

### 4.1. Physiological Data Pre-Processing

For the physiological signals recorded with the Shimmer3 GSR+, Empatica E4, and EmbracePlus, motion artefacts were identified and removed using acceleration data. Specifically, acceleration signals from the three axes (x, y, and z) were first resampled to match the sampling frequency of the corresponding physiological signal. The root mean square (RMS) across the three axes was then computed. A movement threshold was defined as the sum of the mean RMS and its standard deviation, providing a participant-specific and data-driven criterion for identifying periods of elevated movement. Physiological data points with RMS values exceeding this threshold were considered corrupted by motion and were removed. Visual inspection of the processed PPG signals confirmed that this threshold effectively identified motion-contaminated signal segments. Missing segments resulting from this procedure were subsequently interpolated using monotonic cubic interpolation. This interpolation method was chosen to preserve physiologically plausible signal trajectories while avoiding interpolation-induced artificial oscillations.

For the Pixel Watch 2, artefact removal based on acceleration data was not feasible due to the absence of a fixed sampling rate for heart rate measurements. Instead, the device-provided accuracy labels for each sensor event were used for filtering. Only measurements classified as “average” or “maximum” accuracy were retained, while all others were discarded. The resulting gaps in the data were likewise interpolated using monotonic cubic interpolation.

The subsequent processing steps applied after motion artefact removal are described separately for each physiological signal in the following sections.

#### 4.1.1. Cardiological Activity

NeuroKit2 [43] was used for further processing of the interpolated PPG signals obtained from the Shimmer3 GSR+, Empatica E4, and EmbracePlus devices. The signals were first filtered using a third-order Butterworth bandpass filter with cutoff frequencies of 0.05–8 Hz to attenuate baseline drift and high-frequency noise. Subsequently, systolic peaks were detected. Peaks that did not represent physiologically plausible heart rates between 40 and 200 beats per minute (bpm) were filtered out and interpolated. IBIs were then computed from the detected peaks, from which heart rate was derived. To further reduce artefacts, successive IBIs exhibiting changes greater than 40% were excluded. This comparatively lenient threshold, relative to commonly used thresholds of 20–30%, was intentionally chosen to account for the increased noise and variability in typical PPG waveforms, thereby avoiding overly aggressive rejection of valid data. The removed peaks were then reconstructed using NeuroKit2 to create a continuous sequence of peaks for HRV computation. Based on the corrected peak sequences, IBIs were recomputed and subsequently used to derive metrics, including mean heart rate and heart rate variability (HRV) features. These features were calculated separately for each task over a window of 122.5 s, corresponding to the exact duration of each n-back task. For tasks exceeding this duration, a middle time window within each task segment was selected to ensure comparability across conditions. Features for the startle event were calculated over 30 s windows: 5–35 s before the startle honk event, i.e., the end of the three-back task; 1–31 s after the honk event; and the first 30 s of the directly following breathing relaxation phase were taken as windows for the startle event analysis.

Due to the absence of accessible raw PPG data from the Pixel Watch 2, HRV metrics could not be computed. However, the HR values were analyzed.

#### 4.1.2. Electrodermal Activity

After motion artefact removal, the EDA signals from the Shimmer3 GSR+, Empatica E4 and the EmbracePlus were decomposed into their tonic and phasic components using Neurokit2 and the nonnegative sparse deconvolution [44]. Phasic peaks with a minimal amplitude of 0.05 μS were obtained for further analyses. Subsequently, features were calculated for each task and the startle event. The EDA signal of the Pixel Watch 2 was not interpretable and therefore not analyzable.

#### 4.1.3. Skin Temperature

The skin temperature signals from the Empatica E4, EmbracePlus and Pixel Watch 2 were further filtered for physiologically plausible ranges (25–40 °C). As with the PPG and EDA signals, features were subsequently computed for each task and the startle event. Shimmer3 GSR+ did not record skin temperature.

### 4.2. Data Quality

The quality of the physiological signals was assessed by calculating the average percentage of data removed due to motion artefacts or physiologically implausible values (Table 2). The average percentage of data that needed to be removed due to motion artefacts was comparable across devices and signals. The EDA and skin temperature signals from the EmbracePlus were slightly more affected by motion artefacts than those of the Empatica E4. Of the data, 14.1% needed to be additionally removed from the Empatica E4 to ensure that the HR calculated from the BVP signal was within a realistic range. This is more removed data than for Shimmer3 GSR+ (3.9%) and for the EmbracePlus devices (7.8%). The already processed HR and TEMP signals from the Pixel Watch 2 were already within valid ranges (Table 2).

### 4.3. Data Analysis

#### 4.3.1. Friedman Test

The Friedman test was used to analyze the behavioural and subjective data. Error rates, response time, cognitive load, and frustration were compared across n-back tasks using subsequent Wilcoxon post hoc tests and Bonferroni corrections.

#### 4.3.2. Linear Mixed-Effects Models

To assess the comparability of wearables and the reproducibility of physiological measurements across wearables and tasks, we fitted linear mixed-effects models. Analyses were conducted in R using the lme4 package [45]. The dependent variables were the physiological measures. Fixed effects *wearable* (Shimmer3 GSR+, Empatica E4 and Pixel Watch 2 or Empatica E4 and EmbracePlus) and *task* (baseline, one-back, two-back, three-back, relaxation breathing, physical activity), and their interaction (*wearable*:*task*), allowed us to test both main and interaction effects.

To account for the repeated-measures structure, we included the participants as random intercepts. A random slope for physical activity per participant was included, since individual differences in physiological activity are expected to be most pronounced during physical activity (1 + *physical_activity | participant*).

$$physio\;\sim \;wearable+task+wearable:task+\left(1+physical\text{\_}activity\;\right|\;participant)$$

The startle event was analyzed separately from the other tasks due to its short-term, event-based nature. Physiological measures were again treated as dependent variables, with *wearable* (Shimmer3 GSR+, Empatica E4 and Pixel Watch 2 or Empatica E4 and EmbracePlus) and *startle* (before startle, after startle and relaxation) as fixed effects. The interaction effect of the main effects was also considered (*wearable*:*startle*). The random intercept for participants accounted for inter-individual differences.

$$physio\;\sim \;wearable+startle+wearable:startle+\left(1\right|participant)$$

Main effects were evaluated by comparing the full model to reduced models excluding the respective effect using likelihood ratio tests (analysis of variance (ANOVA)). When significant effects were observed, Tukey-corrected post hoc tests were conducted to examine pairwise differences between effect levels.

Model fit of the full and the reduced null models was evaluated using the Akaike Information Criterion (AIC). Lower AIC values indicate a better trade-off between explanatory power and model complexity. Therefore, when comparing AIC values across models, the model with the lower AIC value is considered to provide a better relative fit to the given data.

Model diagnostics prior to analyses indicated right-skewed distributions for several physiological measures, leading to violations of the model assumptions of normality and homoscedasticity. To address this, we applied a logarithmic transformation of the respective measures and used them as the dependent variables. HR, RMSSD, SCL and cleanEDA were log-transformed and will be named lnHR, lnRMSSD, lnSCL, and lnEDA in the following sections.

To assess the sensitivity of the study to detect physiological differences between cognitive load conditions, post hoc simulation-based power analyses were performed using linear mixed-effects models restricted to the three n-back conditions. The models included the fixed effects of *wearable*, *task,* their interaction effect and a random intercept for *participant*. Statistical power was estimated using the simr package in R.

#### 4.3.3. Sensitivity Analyses

Sensitivity analyses were conducted to assess the robustness of the findings with respect to the chosen physiological preprocessing steps. In particular, we investigated whether different amounts of outlier removal between the devices affected the results. As the largest difference in data removal was observed between the Shimmer3 GSR+ and the Empatica E4 for the PPG/HR signal, the sensitivity analysis focused on these devices and this modality.

Three preprocessing pipelines were compared: (1) a minimally processed pipeline including only peak detection on the raw PPG signal, (2) a pipeline including motion artefact removal but omitting the additional IBI/HR outlier removal steps, and (3) the primary preprocessing pipeline described in Section 4, including both motion artefact removal and HR outlier removal. To ensure that differences between pipelines reflected preprocessing rather than differences in the analyzed observations, the comparison was restricted to the subset of observations available across all three preprocessing pipelines.

The same linear mixed-effects models were fitted to each dataset. To facilitate a stable and comparable evaluation across preprocessing pipelines, the sensitivity analyses used a simplified random-effects structure including only participant-specific random intercepts. The participant-specific random slope for physical activity (Section 4.3.2 Linear mixed-effects models), to account for inter-individual differences during physical activity, was omitted because it resulted in singular model fits for some preprocessing pipelines and was not central to the objective of comparing preprocessing strategies. Model fit (AIC) and explanatory power (marginal R^2^) for lnRMSSD were compared to assess the robustness of the findings with respect to the preprocessing strategy. The lnRMSSD metric was selected because it is one of the primary PPG-derived HRV measures analyzed in this study and is directly affected by the pre-processing pipeline.

## 5. Results

### 5.1. Study Design Validation via Behavioural and Subjective Data

During each cognitive n-back task, behavioural performance was assessed using error rates and response times for each participant. Following each n-back task, participants provided subjective ratings of perceived cognitive load and frustration. The startle event was also rated for perceived intensity. Together, behavioural and subjective data give information on experienced task difficulty levels and startle intensity, from which we can deduce whether task- and event-related physiological responses can be expected from our study design. These measures are participant-reported and behavioural outcomes and not wearable-derived measurements. They are therefore not intended as device-comparison outcomes. Instead, they serve to compare task difficulty levels and participant cohorts to validate the cognitive load and startle manipulations.

#### 5.1.1. Cognitive n-Back Tasks

**Behavioural Data: Errors and Response Times**

**Figure 6:**
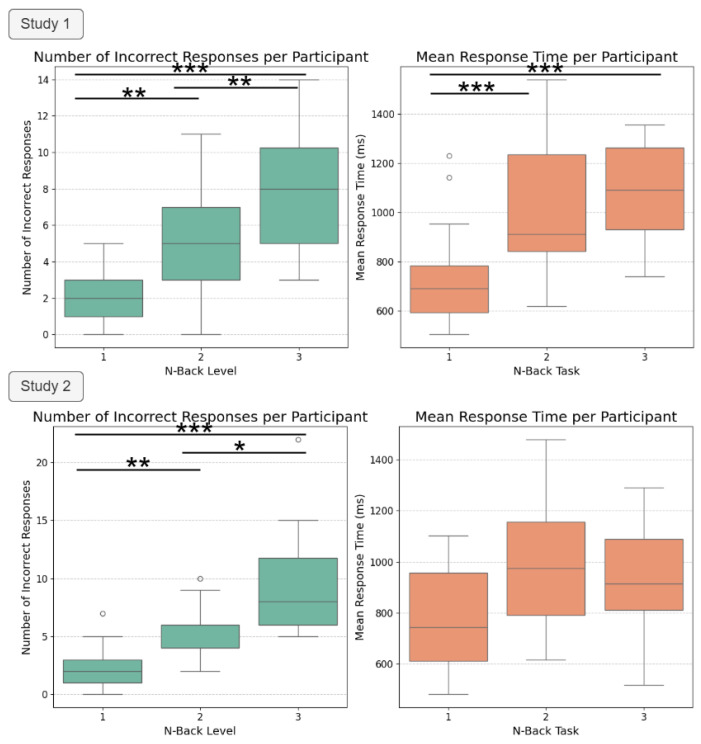
Comparison of incorrect responses and mean response times across n-back task difficulties (1-back, 2-back, 3-back) per participant for study 1 and study 2. Asterisks indicate significant differences with * *p* < 0.05, ** *p* < 0.01 and *** *p* < 0.001.

From a behavioural perspective, task performance and task difficulty can be analyzed by considering the number of errors and the mean response times of the participants during the cognitive n-back tasks (Figure 6). Error rates increased systematically with n-back task difficulty in both studies (Figure 6). Friedman tests confirmed a significant effect of task difficulty on errors (study 1: χ^2^(2) = 29.64, *p* < 0.001; study 2: χ^2^(2) = 19.704, *p* < 0.001). Post hoc Wilcoxon tests with Bonferroni correction revealed significant differences between all n-back levels in both studies (all *p* < 0.05), with medium to large effect sizes (r = 0.65–0.88). These results indicate a robust increase in errors with task difficulty, reflecting increasing cognitive demand across both participant cohorts.

Response times increased with task difficulty in study 1 (Figure 6). A Friedman test indicated a significant effect (χ^2^(2) = 30.90, *p* < 0.001), with post hoc tests showing longer response times in two-back and three-back compared to one-back (both *p* < 0.001, r = 0.88), but no significant difference between two-back and three-back. In study 2, response times showed a similar pattern (Figure 6). However, they did not differ significantly between the n-back tasks (χ^2^(2) = 4, *p* = 0.1353). Overall, for both studies, one could observe that whilst response times increased from one-back to two-back task difficulty, they plateaued at the highest task difficulty (three-back).

**Subjective Data: Perceived Cognitive Load and Frustration**

**Figure 7:**
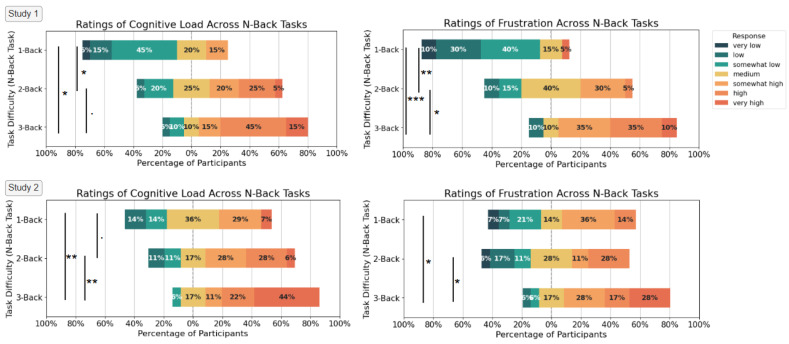
Comparison of cognitive load and frustration ratings across n-back task difficulties (1-back, 2-back, 3-back) per participant for study 1 and study 2. Asterisks indicate significant differences with * *p* < 0.05, ** *p* < 0.01 and *** *p* < 0.001. A dot indicates marginal significance with *p* < 0.1.

Subjective cognitive load increased systematically with n-back task difficulty in both studies (Friedman tests: study 1: χ^2^(2) = 23.46, *p* < 0.001; study 2: χ^2^(2) = 19.48 *p* < 0.001). Post hoc Wilcoxon tests with Bonferroni correction showed almost significantly (*p* < 0.1) to significantly higher perceived cognitive load in three-back compared to one-back and two-back tasks (for all comparisons in study 1 and 2: r ≥ 0.743, *p* < 0.05). Trends were consistent across cohorts (Figure 7), showing large effects of task difficulty on perceived cognitive load.

Frustration ratings followed a similar pattern to cognitive load, increasing with task difficulty (Friedman tests: study 1: χ^2^(2) = 26.41, *p* < 0.001; study 2: χ^2^(2) = 12.04, *p* = 0.002). Post hoc tests indicated significantly higher frustration in three-back versus one-back and two-back conditions (all r ≥ 0.76, *p* < 0.05) in both studies, while significant differences between one-back and two-back were only present in study 1 (W = 14, r = 0.760, *p* = 0.0021) and not in study 2 (W = 17.0, r = 0.596, *p* = 0.7951). Effect sizes indicate strong increases in frustration for the three-back condition in both studies (Figure 7).

#### 5.1.2. Startle Event

**Figure 8:**
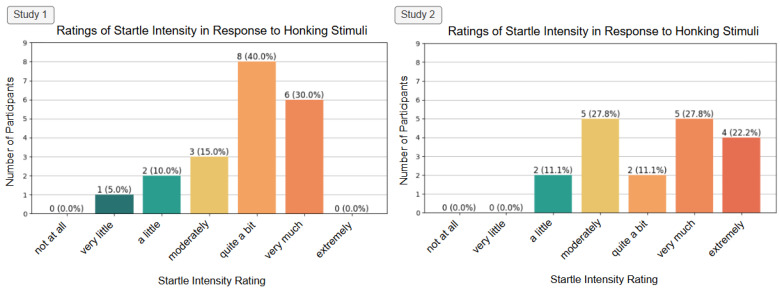
Startle response intensity ratings based on the question: “If you noticed the honking sound, how much did that sound startle you?” for participant cohort of study 1 (**left**) and study 2 (**right**).

In both studies, all participants noticed the honking sound and therefore answered the question: “How much did that sound startle you?” Although the distribution of startle intensity responses differs between the studies, the average startle intensity rating in both participant cohorts is “quite a bit” (Figure 8).

Across both studies, higher n-back difficulty led to more errors and higher perceived cognitive load and frustration, while response times plateaued between the 2-back and 3-back tasks. The honking startle event was perceived by all participants with an average intensity of 5 out of 7. The task design effectively created increasing cognitive load and a clearly noticeable startle event.

### 5.2. Comparability and Reproducibility of Physiological Measurements Across Wearables and Tasks/Events

In this section, we examined whether physiological measures from the Shimmer3 GSR+, Empatica E4 and Pixel Watch 2 differ during individual tasks (baseline, one-back, two-back, three-back, breathing relaxation, physical activity) and whether physiological measures are comparable across tasks. We fitted linear mixed-effects models to predict each physiological measure. First, we assessed the significance of the wearable–task interaction on the model fit to determine whether differences between wearables depend on the tasks. When the interaction was significant, post hoc tests were conducted to identify, for each task, which devices differed and, for each device, which tasks elicited different physiological responses. If the interaction was not significant, the main effects of *wearable* and *task* were analyzed individually to determine whether physiological measures differed between wearables overall and across tasks, respectively.

#### 5.2.1. Sensitivity Analysis of Pre-Processing Pipelines

To assess the influence of physiological preprocessing on the statistical analyses, the linear mixed-effects model for lnRMSSD was repeated using the three preprocessing pipelines described in Section 4.3.3 Sensitivity Analysis. The results are summarized in Table 3. To isolate the effect of preprocessing from differences in the analyzed observations, model comparisons were restricted to the subset of tasks available across all three preprocessing pipelines (N_tasks_ = 228).

The minimally processed pipeline (P_minimal_) consistently showed the poorest performance, with the poorest model fit and the lowest explained variance (AIC = 577.0; marginal R^2^ = 0.203). Although the preprocessing pipeline, including motion artefact removal without additional IBI/HR outlier removal (P_motion_), achieved the best model fit (AIC = 476.9), the P_motion+HR_ pipeline yielded the highest marginal R^2^ (0.442) while additionally excluding physiologically implausible HR changes. Furthermore, the direction of the estimated device and task effects remained consistent across all preprocessing pipelines, indicating that preprocessing primarily influenced statistical model fit rather than the overall physiological interpretation. Therefore, the _Pmotion+HR_ was used for the subsequent analyses because it provided the best balance between model fit, explanatory power and physiologically meaningful HRV estimation.

#### 5.2.2. Interaction Effect Between Wearables and Tasks

AIC values of the full model, which included the interaction effect between wearables and tasks, and the null model, which excluded the interaction effect, were compared to evaluate model fit. Lower AIC values for the full model, together with a significant likelihood-ratio test, indicate that including the interaction effect improves the model’s fit to the data.

**Figure 9:**
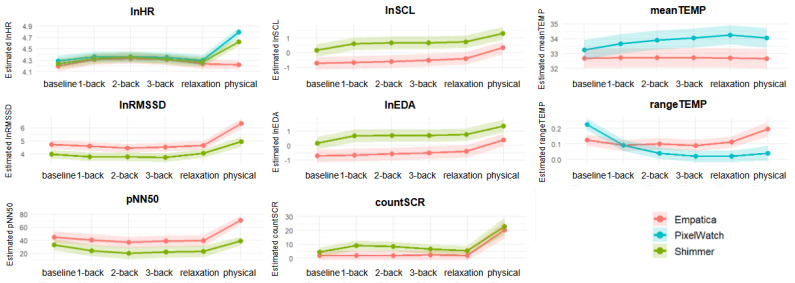
Interaction plots per physiological measure to visualize the wearable:task interaction effect. The tasks are depicted on the x-axis, and the wearables are displayed as coloured lines. The colour shadings around the lines indicate the 95% confidence intervals.

No significant interaction effects between wearables and tasks were observed for the EDA measures, including log-transformed mean SCL (lnSCL), log-transformed combined EDA (lnEDA), and SCR count (countSCR), as indicated by likelihood ratio tests comparing full models to null models without the interaction effect (all *p* > 0.53; Table 4). In contrast, log-transformed heart rate (lnHR), heart rate variability measures (lnRMSSD, pNN50), and temperature measures (meanTEMP, rangeTEMP) showed significant interaction effects (all *p* < 0.05), indicating that the differences in physiological responses between wearables depend on the task performed. Interaction plots in Figure 9 illustrate how wearable differences vary across tasks, with crossing or diverging lines highlighting the task-dependent nature of the physiological signals captured by the wearables. Post hoc tests were then conducted to identify specific device and task differences driving these interactions for cardiological and skin temperature measures (see Section 5.2.3 PPG and TEMP—Interaction Effect (wearable:task) Analysis), while the main effects were analyzed for the electrodermal activity measures (see Section 5.2.4 EDA—Analysis of the Main Effects Wearable and Task).

#### 5.2.3. PPG and TEMP—Interaction Effect (wearable:task) Analysis

**Reproducibility of Physiological Measures Per Task (Device Differences Per Task)**

In the following, we examine whether physiological measures from wearables differ across the individual tasks.

**HR (lnHR).** Significant interaction effects between wearable and task were observed. During the baseline task, the Empatica E4 recorded significantly lower lnHR compared to the Pixel Watch 2 (*p* = 0.049). No device differences emerged during the n-back tasks or breathing relaxation phase. However, during physical activity, all devices differed from each other: the Empatica E4 showed significantly lower lnHR than both the Pixel Watch 2 and the Shimmer3 GSR+ (both *p* < 0.001), and the Shimmer3 GSR+ recorded significantly lower values than the Pixel Watch 2 (*p* < 0.001). Especially during physical activities, the wearables differ greatly in their measured heart rate. These results indicate substantial divergence in HR measurements during physical activity (Figure 9, lnHR).

**HRV (lnRMSSD, pNN50).** Systematic device differences were found for both HRV metrics lnRMSSD and pNN50. Across all tasks, Empatica E4 produced significantly higher HRV values than the Shimmer3 GSR+ (all *p* < 0.0037) (Figure 9, lnRMSSD, pNN50).

**Skin temperature (meanTEMP, rangeTEMP).** Pixel Watch 2 consistently reported higher meanTEMP values than Empatica E4 across all tasks (all *p* < 0.001) (Figure 9, meanTEMP). For rangeTEMP, Empatica E4 showed lower values than Pixel Watch 2 during the baseline task (*p* < 0.0001), no differences during one-back, and higher values during two-back, three-back and physical tasks (all *p* < 0.015) (Figure 9, rangeTEMP).

**Comparability of Devices’ Physiological Measures Across Tasks (Task Differences Across Devices)**

Next, we consider whether the wearables capture similar physiological trends across tasks.

**Figure 10:**
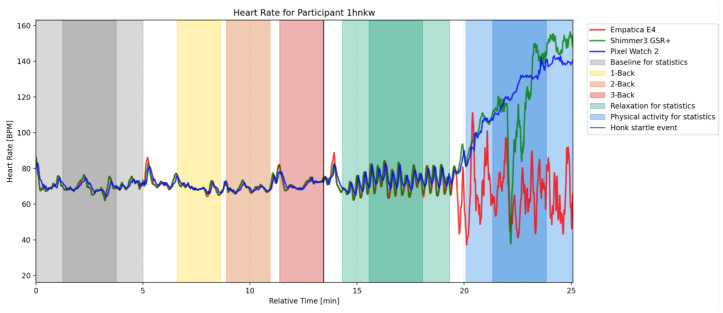
A participant’s heart rate across the tasks of study 1 for the three wearable devices, Empatica E4 (red line), Shimmer3 GSR+ (green line) and Pixel Watch 2 (blue line), is depicted. The different study phases are marked in the background. For longer tasks, the middle (darker) time sections were considered for statistical analyses.

**HR (lnHR).** Shimmer3 GSR+ and Pixel Watch 2 both showed higher lnHR during physical activity compared to all other tasks (all *p* < 0.001). In contrast, Empatica E4 showed limited sensitivity to task differences, with only baseline vs. two-back reaching significance (*p* = 0.008). Thus, Shimmer3 GSR+ and Pixel Watch 2 captured expected HR increases during physical activity, whereas the Empatica E4 did not (Figure 9, lnHR). The HR signals of the devices across the study tasks for one participant are depicted in Figure 10. Whilst strong similarities can be observed across devices and tasks, a strong divergence between the signals and their quality can be seen during physical activity.

**HRV (lnRMSSD, pNN50).** For lnRMSSD, Shimmer3 GSR+ and Empatica E4 showed similar activity patterns, with higher values during physical activity compared to baseline and one-back conditions, but lower values during two-back, three-back, and relaxation (Figure 9, lnRMSSD). For pNN50, Empatica E4 showed consistently higher values during physical activity compared to all other tasks (all *p* < 0.001). Shimmer3 GSR+ showed limited differentiation. Only higher pNN50 values could be observed for the physical activity compared to the two-back task (*p* = 0.0172) and between the baseline and two-back task (*p* = 0.0319) (Figure 9, pNN50). In sum, lnRMSSD trends align across devices, but pNN50 patterns diverge.

**Skin temperature (meanTEMP, rangeTEMP**). Empatica E4 showed no task-related differences in meanTEMP. In contrast, Pixel Watch 2 detected higher temperatures during physical activity, relaxation, and higher cognitive load (two-back, three-back) relative to baseline (all *p* < 0.001) and also lower values during one-back compared to relaxation (Figure 9, meanTEMP). For Empatica E4, differences in rangeTEMP were modest and primarily involved physical activity, with larger ranges during physical activity than in the one-back, three-back, and relaxation conditions (all *p* < 0.038). Pixel Watch 2 showed significantly higher rangeTEMP during the baseline condition compared to all other tasks (all *p* < 0.001). One-back also had a higher range than the two-back and the relaxation conditions (*p* < 0.0438) (Figure 9, rangeTEMP). In sum, Empatica E4 and the Pixel Watch differed strongly in temperature measurements across tasks.

#### 5.2.4. EDA—Analysis of the Main Effects Wearable and Task

For the three EDA measures, no significant interaction effect between tasks and wearables could be observed and the AIC indicated a better model fit without considering the interaction effect (Table 4, Figure 9). Therefore, the main effects of *wearable* and *task* were analyzed separately.

Across all three EDA measures, significant differences between devices were found: the Shimmer3 GSR+ consistently produced higher values than the Empatica E4 for lnSCL (*p* < 0.001), lnEDA (*p* < 0.001) and number of SCRs (*p* < 0.001) (Table 5, Figure 9).

The main effect of *task* was also significant for all measures (lnSCL: *p* < 0.001; lnEDA: *p* < 0.001, countSCR: *p* = 0.0015) (Table 5). Post hoc comparisons showed that physical activity elicited significantly larger responses compared to all other tasks across all three EDA measures (all *p* < 0.05). Differences across the cognitive load and relaxation tasks were minor and not statistically significant across the three EDA measures.

#### 5.2.5. Startle Event—Physiological Comparison Across Wearables

**HR (lnHR).** No significant interaction effect between wearables and the startle phases was found. However, the wearables differed significantly from each other (Table 6), with the Pixel Watch 2 reporting significantly higher values than both the Empatica E4 (*p* < 0.001) and the Shimmer3 GSR+ (*p* = 0.0022).

**Figure 11:**
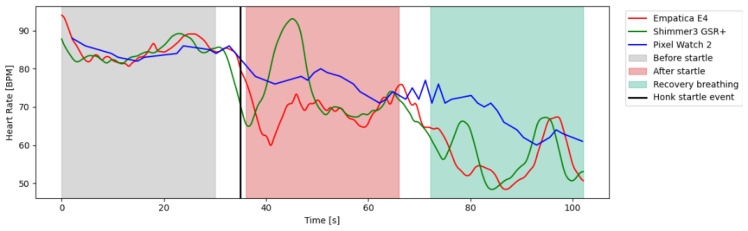
A participant’s heart rate as retrieved from the Empatica E4 (blue), the Shimmer3 GSR+ (red) and the Pixel Watch 2 (green) are depicted as lines. The honking startle event is marked with a black vertical line. The startle phases before the startle event (grey), after the startle event (red) and during the breathing recovery phase (green) are marked in the background.

A significant main effect of the startle phase was also found (Table 6). Pairwise comparisons showed no significant difference in lnHR between before and after the startle event (*p* = 0.6578). In contrast, lnHR values during the recovery breathing phase were significantly lower than during the before-event (*p* < 0.001) and after-event phase (*p* = 0.0022). An illustrative example of the characteristic decrease followed by an increase in heart rate following the startle event is depicted in Figure 11, with varying clarity across wearables.

**HRV (lnRMSSD, pNN50).** For both HRV metrics, no significant interaction effect was observed. However, significant differences between the wearables were found (Table 6), with the Empatica E4 having higher lnRMSSD and pNN50 values than the Shimmer3 GSR+ (both *p* < 0.001).

The startle phases differ significantly regarding their lnRMSSD and pNN50 values (Table 6). Pairwise comparisons showed that lnRMSSD and pNN50 were significantly higher in the after-startle phase compared to the before-startle phase (p_lnRMSSD_ = 0.0016, p_pNN50_ = 0.0183). Similarly, both metrics were higher during the recovery breathing phase than before the startle event (both *p* < 0.001). No significant differences were observed between after the event and the breathing phase (p_lnRMSSD_ = 0.3619, p_pNN50_ = 0.3614).

**EDA (lnSCL, lnEDA, countSCR).** Across all three EDA measures, no significant interaction effect was present, but significant differences between wearables were observed (Table 6). Shimmer3 GSR+ measured higher values than the Empatica E4 (*p* < 0.001).

**Figure 12:**
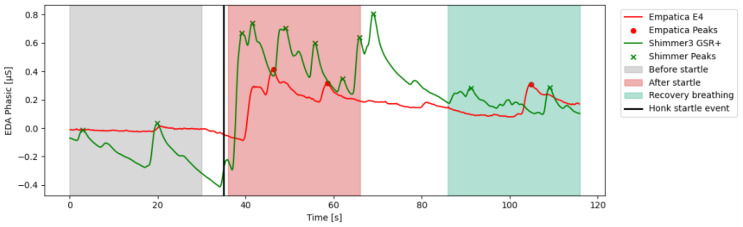
The phasic component of the cleaned EDA signal with the phasic peaks of one participant is depicted. SCRs are marked for Empatica E4 (blue) with dots and for Shimmer3 GSR+ (red) with crosses. The honking startle event is marked with a black vertical line. The three startle phases before the startle event (grey), after the startle event (red) and during the recovery breathing phase (green) are marked in the background.

No significant differences between startle phases were found for lnSCL and lnEDA (Table 6). However, the number of SCRs increased significantly after the startle event compared to before (*p* < 0.001) and was significantly higher than during the relaxation breathing phase (*p* = 0.0475). The phasic component of the cleaned EDA signal with SCRs is shown for one participant in Figure 12.

**Skin temperature (meanTEMP, rangeTEMP).** The skin temperature between Empatica E4 and Pixel Watch 2 could not be compared reliably due to the different data sampling methods of the wearables (Empatica E4: fixed sampling rate; Pixel Watch 2: event-based). Not enough data points for the Pixel Watch 2 were recorded during the short startle event phases for calculating the metrics and comparing them with those of the Empatica E4.

Wearables showed substantial differences in absolute physiological values, but captured similar trends, particularly for strong effects such as physical activity and startle responses. Overall, signals were consistent under low-motion conditions but diverged during physical activity.

### 5.3. Empatica E4 vs. EmbracePlus

In this section, the physiological measures of the outdated Empatica E4 and the follow-up model EmbracePlus wristband are compared. The data for each wearable were collected from separate study cohorts. Only the n-back tasks, the startle event and the relaxation breathing task were included in study 2 using the EmbracePlus, which is why the present comparison focuses on these tasks. In the following, we first analyze the cognitive n-back and relaxation breathing tasks, followed by the honk startle event.

#### 5.3.1. Cognitive n-Back and Breathing Relaxation Tasks

**HR (lnHR).** No interaction effect or significant main effect of *wearable* was observed for lnHR, but the main effect of *task* was significant (Table 7). Post hoc comparisons indicated that the three n-back tasks did not differ significantly from each other. However, compared to the relaxation breathing phase, lnHR was significantly higher during the two-back task (*p* = 0.0096) and also tended to be higher for the three-back task (*p* = 0.0603). These findings indicate that lnHR was elevated during higher cognitive load conditions compared to the subsequent relaxation breathing phase.

**HRV (lnRMSSD, pNN50)**. No significant main effects of *wearable* or *task* and no significant interaction effects were observed for lnRMSSD or pNN50 (Table 7). This suggests comparable measurements between devices and no evidence of task-related changes in these HRV indices.

**EDA (lnSCL, lnEDA, countSCR).** No interaction effect was found for the three EDA metrics (Table 5). A significant main effect of *wearable* was observed for both lnSCL and lnEDA (Table 5), with the Empatica E4 yielding higher values than the EmbracePlus (both *p* < 0.001). A significant main effect of *task* was also found for both metrics (both *p* < 0.001) (Table 5). Higher values were recorded during the relaxation breathing task compared to the one-back (*p* < 0.001), two-back (*p* < 0.001) and three-back (p_lnSCL_ = 0.0566, p_lnEDA_ = 0.0591). The number of SCRs differed significantly between the two devices, with the EmbracePlus detecting on average 45.6 more peaks than Empatica E4 (*p* < 0.001). No significant differences in SCR count were found across task conditions (Table 7).

**Skin temperature (meanTEMP, rangeTEMP).** No significant interaction effect and no significant main effect of *wearable* were observed for meanTEMP or rangeTEMP (Table 7). A significant main effect of *task* was found for rangeTEMP, but not for meanTEMP. Post hoc comparisons indicated that the temperature range was significantly higher during the relaxation breathing task compared to the n-back tasks (p_1-back_ = 0.0320, p_2-back_ = 0.0165, p_3-back_ = 0.0170).

#### 5.3.2. Startle Event—Embrace E4 vs. EmbracePlus

**HR (lnHR).** A significant interaction effect between wearable and startle phase was observed for lnHR (*p* = 0.0408) (Table 8). Post hoc analyses indicated no significant differences between devices within any of the individual startle phases. The Empatica recorded significantly lower lnHR during the breathing recovery phase compared to before (*p* < 0.001) and after the startle event (*p* = 0.0084). No significant difference between before and after the startle event could be observed. In contrast, the EmbracePlus did not record significant differences in lnHR between the three startle phases.

**HRV (lnRMSSD, pNN50).** The interaction effect for lnRMSSD was not significant and neither was the main effect of *wearable* (Table 8). However, a significant main effect of *startle* was observed. lnRMSSD was significantly higher after the startle event compared to before the event (*p* = 0.0157) and also higher during the recovery phase than before the event (*p* = 0.0235). No significant difference was found between the post-startle and recovery phases.

For pNN50, the interaction effect was significant. Post hoc analyses revealed a significant device difference during the pre-startle phase, with the EmbracePlus showing higher values than the Empatica E4 (*p* = 0.0134). No significant device differences were found during the post-startle or recovery phases. For the Empatica E4, pNN50 was significantly higher after the startle event than before the event (*p* = 0.0043) and higher during the recovery breathing phase compared to before the event (*p* < 0.001). No significant differences between startle phases could be observed for the EmbracePlus.

**EDA (lnSCL, lnEDA, countSCR).** No interaction effect was observed for lnSCL and lnEDA (Table 8). The devices differed significantly for lnSCL and lnEDA, with Empatica recording higher values than EmbracePlus (p_lnSCL_ = 0.001, p_lnEDA_ < 0.001). Additionally, higher skin conductance levels were recorded during the recovery phase compared to before the event (p_lnSCL_ < 0.001, p_lnEDA_ = 0.0011), whilst no further significant differences between startle phases were observed.

An interaction effect between *wearable* and *startle* could be observed for countSCR. During all three startle phases, EmbracePlus recorded 9–11 more SCRs than Empatica (all *p* < 0.001). No significant differences between startle phases could be observed for the Empatica E4. In contrast, EmbracePlus recorded on average 1.316 more SCRs during the recovery phase compared to directly after the event (*p* = 0.0248).

**Skin temperature (meanTEMP, rangeTEMP).** For the skin temperature metrics meanTEMP and rangeTEMP, neither the interaction effect nor the main effects were significant (Table 8).

Task-related physiological changes are captured similarly by the Empatica E4 and EmbracePlus, but differences in sensitivity to short-term event-related changes are evident.

### 5.4. Device Comparison Regarding Comfort

**Figure 13:**
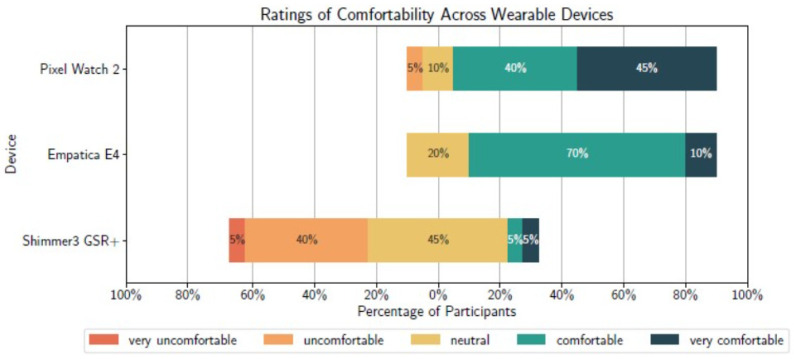
Comfortability ratings across wearable devices (Google PixelWatch 2, Empatica E4, and Shimmer3 GSR+) based on the question, “How would you rate the comfortability of the device?” (study 1).

In study 1, the Google Pixel Watch 2 received predominantly positive comfort ratings, as illustrated in Figure 13. Of the 20 participants, nine rated it as very comfortable and eight as comfortable, resulting in 17 participants (85%) reporting a positive experience. Two participants provided neutral ratings, while one participant rated the device as uncomfortable.

The Empatica E4 yielded similar results, with two participants rating it as very comfortable and 14 as comfortable, totaling 16 positive ratings (80%). Four participants reported neutral experiences, and no negative ratings were observed.

In contrast, the Shimmer3 GSR+ received comparatively lower comfort ratings. Only one participant rated it as very comfortable and one as comfortable. Nine participants reported neutral experiences, while nine participants indicated negative perceptions, including eight ratings of uncomfortable and one of very uncomfortable.

In study 2, participants were asked in an open-ended question whether the wristband (EmbracePlus) disturbed them during their work routine and, if so, in which situations. Of 18 participants, 16 (88.89%) reported no disturbance, with some explaining that they were accustomed to wearing watches or fitness trackers and barely noticed it. One participant reported that the device was mostly unobtrusive during work, but it sometimes disturbed them during personal tasks such as exercising or showering. One participant did not answer the question. Overall, high comfort due to no or very little disturbance can be inferred.

Most participants found the Pixel Watch 2, Empatica E4, and EmbracePlus comfortable or unobtrusive, while the Shimmer3 GSR+ was often rated as uncomfortable.

## 6. Discussion

### 6.1. RQ1: Effective Study Design

The two participant cohorts of study 1 and study 2 showed similar behavioural patterns during the cognitive n-back tasks: (1) error rates increased with task difficulty, and (2) response times increased more strongly from one-back to two-back but plateaued between two-back and three-back. The continued rise in errors alongside stable response times at the highest difficulty level suggests that participants reached a cognitive load limit during the three-back task, as they could no longer process the task any faster. Rather than slowing down, participants maintained response speed at the cost of response accuracy.

Consistent trends across studies were also observed in subjective ratings. Perceived cognitive load increased systematically with task difficulty, reaching its highest level in the three-back condition. Frustration ratings followed a similar pattern in study 1. However, in study 2, frustration did not differ significantly between the one-back and two-back conditions. Participants in study 2 reported higher absolute frustration during the one-back task than those in study 1. These findings suggest that initial difficulties in understanding or adapting to the n-back task may account for the observed differences in ratings between the participant cohorts.

Regarding the startle event, all participants detected the honking sound, with an average intensity rating of 5 out of 7 across both cohorts, despite some variability in response distributions. This suggests that the honking stimulus was consistently salient.

In sum, the subjective and behavioural data across studies suggest that observable task- and event-related changes in physiological responses can be expected. The results support the validity and effectiveness of the study design.

### 6.2. RQ2: Comparability and Reproducibility of Physiological Measures Across Research- and Consumer-Grade Wearables

The aim of this work was to investigate how comparable and reproducible physiological measurements from research-grade and consumer wearables are in capturing task- and event-related physiological responses. Overall, the results show that while devices often agree on physiological trends across tasks, they differ substantially in absolute values and in their robustness under certain conditions, particularly during movement. Sensitivity analyses indicated that pre-processing primarily affected statistical model fit rather than the interpretation of the physiological responses.

#### 6.2.1. Cardiological Measures

Heart rate measurements were largely consistent across devices under low-motion conditions (baseline, cognitive tasks, and relaxation) but diverged strongly during physical activity. The Pixel Watch 2 reported the highest values, followed by Shimmer3 GSR+, while the Empatica E4 yielded the lowest values. These results suggest that motion plays a critical role in signal quality and resulting heart rate estimates.

Although extreme motion artefacts were removed during preprocessing of the physiological signals, residual artefacts persisted in the PPG signals. This was particularly evident for the Empatica E4 and Shimmer3 GSR+, where increased signal variance and noise were observed. For the Empatica E4, a relatively large proportion of PPG data had to be excluded due to motion artefacts and implausible values. While missing segments were interpolated, the resulting signal likely remained unreliable, as reflected by the strong signal variance (example data from one participant in Figure 10). In contrast, the Pixel Watch 2 showed a smoother and more gradual increase in heart rate during physical activity, consistent with expected physiological responses. However, due to the lack of transparency of its internal preprocessing, it remains unclear whether this stability reflects effective artefact handling, or algorithmic smoothing or estimation.

Differences in sensor placement may further explain these observed discrepancies. The Shimmer3 GSR+ records PPG signals at the fingers, whereas the Empatica E4 and the Pixel Watch 2 measure PPG at the wrist. These different placements coincide with differences in vascular characteristics, tissue composition and susceptibility to motion artefacts. To reduce differences between devices regarding motion artefacts, which were expected to be strongest during physical activity, participants rested their hands on the back of a chair while jogging. This likely resulted in greater wrist movement relative to finger movement and may therefore partly explain the higher proportion of motion artefacts and data exclusion observed with wrist-worn devices compared to the Shimmer3 GSR+.

For HRV, a notable and counterintuitive finding was that lnRMSSD values were higher during physical activity than during baseline and low-cognitive-load conditions. This contrasts with the expected decrease in HRV under physical exertion due to sympathetic activation and parasympathetic deactivation [46]. This discrepancy is likely attributable, at least in part, to motion-related artefacts inflating beat-to-beat variability.

The divergence between lnRMSSD and pNN50 indicates that HRV metrics differ in sensitivity under different measurement conditions. While lnRMSSD showed relatively consistent trends for both Shimmer3 GSR+ and Empatica E4, pNN50 exhibited greater variability and less consistent task differentiation. As pNN50 is based on the proportion of large inter-beat intervals, it is more sensitive to the distribution of large fluctuations, such as those that can be induced by motion artefacts. lnRMSSD, as a continuous measure, appears to be a more robust HRV metric, especially under movement conditions. These results are consistent with prior work [22], which reported limited agreement of HRV measurements from PPG-based wearables and ECG, particularly under non-rest and movement conditions.

During the evaluation of the startle event phases (before the event, after the event and during the breathing relaxation phase), no interaction effects were observed for cardiological metrics, indicating that devices showed comparable relative changes across phases. Heart rate was lowest during the relaxation breathing phase compared with before and after the startle event, consistent with expected physiological responses. The lack of significant differences between the pre- and post-event phases can be partly explained by carry-over effects from the cognitively demanding three-back task, as the pre-event phase corresponds to its final 30 s, during which elevated heart rate due to cognitive processing is expected. In addition, typical startle-related heart rate responses occur over short time scales, involving a brief initial decrease followed by a rapid increase within a few seconds [47]. The use of a 30 s analysis window, chosen to account for potential sensor-specific time lags and to ensure capture of the event, likely averaged out these transient changes.

This interpretation is supported by the HRV results. Both lnRMSSD and pNN50 were significantly higher during the relaxation phase compared to before the event. This aligns with expectations, as the cognitively demanding three-back task before the startle tends to reduce HRV, whereas relaxation increases it. Although an immediate post-startle decrease in HRV would be expected as a stress response, the 30 s analysis window likely averaged out short-term fluctuations. Specifically, the 30 s window likely smoothed these short-term effects, limiting the sensitivity of both metrics to brief physiological responses.

#### 6.2.2. Electrodermal Activity

The EDA results showed consistent and significant main effects of device and task but no significant interaction between the two. While the Shimmer3 GSR+ produced systematically higher values than the Empatica E4 across all measures, both devices exhibited similar patterns across tasks. In particular, physical activity elicited significantly higher EDA responses than all other conditions for both wearables. The absence of an interaction effect indicates that, despite differences in absolute signal magnitude, the devices are comparable in their ability to capture relative changes across conditions.

The systematic offset between devices is likely attributable to differences in sensor placement [21]. The Shimmer3 GSR+ uses finger-based electrodes, which capture stronger electrodermal responses due to higher sweat gland density [48]. Consequently, higher absolute conductance values are expected independent of the device itself. In contrast, the wrist-worn Empatica E4 is more susceptible to lower signal amplitudes, which explains lower SCL, a lower overall EDA signal, and fewer detected SCRs.

During the evaluation of the startle event phases, no interaction effects for the electrodermal metrics were found, supporting the above findings that indicate comparable relative changes during the startle phases across wearables. The tonic EDA measures lnSCL and lnEDA did not capture significant differences across startle phases. This is expected as these features reflect slower changes in skin conductance and are less sensitive to short-term event-related events. In contrast, the SCRs are highly event-related and capture short-term effects. In contrast, the phasic measure countSCRs captured event-related dynamics, with more SCRs observed after the event compared to before the event and during relaxation. This pattern aligns with the expected sympathetic activation elicited by a startle response.

#### 6.2.3. Skin Temperature

The skin temperature measurements revealed device- and task-dependent differences. Across all tasks, the Pixel Watch 2 consistently reported higher mean temperatures than the Empatica E4. Additionally, rangeTEMP patterns differed between the devices: the Empatica E4 showed a lower range at baseline, comparable values during the one-back task, and a higher range during higher cognitive load and physical activity. These results can be explained via the wearables’ different data sampling methods. Whilst Empatica E4 samples data continuously at 4 Hz, the Pixel Watch 2 samples event-dependently. Consequently, the Empatica E4 is better suited for detecting subtle temperature changes, whereas the Pixel Watch 2 provides a more stable but less detailed representation of temperature dynamics.

#### 6.2.4. Implications for Wearable-Based Research

Taken together, these findings highlight key trade-offs between consumer- and research-grade wearables. For HR, the consumer-grade device Pixel Watch 2 appears more robust to motion, likely due to internal artefact handling or smoothing. Because raw PPG data were not interpretable, this assessment is limited to the processed heart rate output provided by the device. It is therefore suitable for empirical studies focusing on heart rate analysis in dynamic settings, provided that HRV metrics and fixed sampling rates are not required and that researchers are willing to implement custom data access solutions. In contrast, research-grade devices such as Empatica E4 and Shimmer3 GSR+ provide access to raw data, enabling transparent signal preprocessing and HRV analysis. However, when used in mobile or real-world conditions, advanced motion artefact removal methods are necessary. Among HRV metrics, RMSSD appears more robust than pNN50 under movement conditions and should be preferred in such setups.

For electrodermal activity, only the research-grade devices provided access to the raw EDA signals. Both the Shimmer3 GSR+ and the Empatica E4 reliably captured increases in EDA during physical activity and event-related reactions via SCRs. Higher sampling rates, higher absolute values, and more detected SCR peaks are advantages of the Shimmer3 GSR+, which enable the calculation of additional metrics for the phasic signal component, e.g., the amplitude and recovery time of the SCRs. The more sparsely detected SCRs and low absolute values would make these analyses more challenging for the Empatica E4.

Skin temperature shows limited comparability across devices due to differences in sampling strategies—Empatica E4 at a fixed sampling rate and the Pixel Watch 2 in an event-based fashion. As temperature changes occur over relatively long time scales, both consumer- and research-grade devices are generally suitable. However, fixed sampling rates facilitate multimodal signal alignment and offer the possibility to analyze temperature changes more precisely.

Overall, reproducibility across devices is limited for absolute values but remains acceptable for relative changes across conditions.

### 6.3. RQ3: Empatica E4 vs. EmbracePlus

The Empatica E4 was previously a widely used research-grade wristband. It is now outdated and was replaced by the EmbracePlus wristband. The question remains how comparable the devices are and whether the new EmbracePlus wristband can reproduce the physiological measures of the Empatica E4. Because the EmbracePlus was evaluated on a separate study cohort and was not worn simultaneously with the Empatica E4, this comparison should be regarded as exploratory.

#### 6.3.1. Cardiological Measures

The exploratory comparison suggests that both wearables capture the heart rate and HRV dynamics across tasks in a comparable manner. The lnHR measure differentiated between higher cognitive load levels and relaxation, rather than between levels of cognitive load within the n-back task. As expected, heart rate was higher during higher cognitive load tasks compared to the relaxation phase. Contrary to expectations, neither device detected HRV differences between the cognitive load and relaxation tasks. A plausible explanation for the results could be the higher sensitivity of time-domain HRV metrics (lnRMSSD and pNN50) to PPG signal quality and preprocessing compared to mean heart rate. In particular, motion artefact correction and subsequent interpolation of inter-beat intervals may reduce short-term signal variability, thereby attenuating subtle task-related differences in HRV while leaving average heart rate largely unaffected. Wrist movements were likely higher during the n-back task than during the relaxation task, which may have led to greater signal correction during the n-back task and thereby reduced the detectable HRV differences between tasks.

During the startle event phases (before the event, after the event and during the breathing relaxation phase), an interaction effect for lnHR was observed. Whilst Empatica E4 showed startle phase changes (see discussion above), the EmbracePlus did not show significant differences between startle phases. These results could point towards a reduced sensitivity of EmbracePlus to short-term HR fluctuations. However, cohort-related differences cannot be excluded as an alternative explanation. The higher lnRMSSD values after the startle event and during the relaxation phase compared to before the event align across Empatica E4 and EmbracePlus, as well as with the Shimmer3 GSR+ device, as described in the previous research question. pNN50 showed greater device-dependent variability but may also not be the most robust measure, as discussed previously in detail.

#### 6.3.2. Electrodermal Measures

EDA results showed differences in devices measuring tonic and phasic activity. Higher tonic activity was recorded by the Empatica E4, whereas the EmbracePlus detected more SCRs. Despite these systematic differences, the exploratory comparison suggests that both devices captured similar task-related effects in tonic EDA, with higher values during the relaxation breathing phase compared to each of the n-back conditions. Elevated skin conductance level has been reported for controlled breathing [49] and seems to be higher than skin conductance induced by cognitive load in this study design. No task-related differences were observed for the phasic SCRs. This finding is consistent with SCRs as event-driven responses, which are typically more sensitive to discrete, salient stimuli than to sustained cognitive or relaxation states.

As discussed for the previous research question, tonic skin conductance measures are more reliable for longer time-periods whilst the SCRs are event-related. For the startle event, we therefore focus our discussion on the SCRs. Within the present datasets from both studies and as already observed during the tasks, the EmbracePlus detected significantly more SCRs than the Empatica E4. Whilst Empatica did not uncover any startle-phase-related differences in the number of SCRs, the EmbracePlus detected slightly more peaks (1–2 peaks) during the recovery phase compared to after the event. This result could be due to differences in peak-detection sensitivity, with the EmbracePlus potentially also capturing more ambiguous fluctuations in the EDA signal.

#### 6.3.3. Skin Temperature

The exploratory comparison did not reveal systematic differences in skin temperature measurements between the Empatica E4 and EmbracePlus. While mean skin temperature remained stable across tasks, temperature variability (rangeTEMP) increased during relaxation breathing. This result could reflect changes in peripheral vasomotor regulation associated with relaxed states and rhythmic breathing, which can induce dynamic fluctuations in peripheral blood flow, leading to increased skin temperature [49].

During the startle phases, neither the wearables nor the task differed in regard to the temperature metrics. Since skin temperature changes slowly, an event-related response is also not expected.

#### 6.3.4. Take-Away of the Comparison

In sum, the Empatica E4 and EmbracePlus capture task-related physiological changes in a largely comparable manner, particularly for HRV and skin temperature. However, differences emerge in sensitivity to short-term dynamics and absolute signal characteristics. Based on our results, Empatica was more sensitive to event-based fluctuations in HR than the EmbracePlus, while the EmbracePlus detects a higher number of event-related SCRs. Despite these discrepancies, both devices show consistent relative changes across tasks, indicating good comparability but limited agreement in absolute values. Overall, the exploratory comparison suggests comparable physiological response patterns between the Empatica E4 and EmbracePlus. Differences in short-term HR dynamics and SCR detection should be interpreted with caution because the devices were evaluated in separate participant cohorts.

### 6.4. RQ2 and RQ3: Methodological Considerations

Based on the behavioural and subjective data, corresponding physiological differences between the cognitive n-back levels would be expected. However, none of the investigated wearables and physiological metrics consistently differentiated between the three cognitive load levels. One explanation may be that the physiological differences associated with increasing cognitive load were comparatively subtle relative to the inter-individual variability and measurement noise inherent in wrist-worn physiological sensing. This interpretation is further supported by the post hoc simulation-based power analyses, which estimated statistical power of 32.7% for study 1 and 26.6% for study 2 to detect physiological differences between the n-back conditions, indicating limited sensitivity to detect such subtle effects.

### 6.5. RQ4: Comfort of Wearing

The wearable devices differed in comfort and unobtrusiveness ratings. The Pixel Watch 2, Empatica E4 and EmbracePlus received predominantly positive ratings, whilst nearly half of the participants in study 1 rated the Shimmer3 GSR+ as uncomfortable. This difference is likely attributable to the sensor placement of the Shimmer3 GSR+, which requires electrodes to be attached to the fingers and may interfere with natural hand movements. In contrast, the Empatica E4 and EmbracePlus position sensors on the wrist, allowing for less intrusive data collection during everyday activities.

The EmbracePlus was perceived as unobtrusive, which may be related to its slim design mimicking strong consumer-grade smartwatches. Consumer wearables such as the smartwatch Pixel Watch 2 are specifically designed for everyday wear and comfort, which explains its overall highest comfort ratings among the four wearables.

Overall, these findings highlight the importance of device design and sensor placement for user comfort, particularly in studies requiring prolonged wear and data collection. Consumer-oriented smartwatches and well-designed research wearables can minimize interference with daily activities, thereby supporting reliable physiological measurements.

## 7. Considerations and Future Work

The comparison of the Empatica E4 and the EmbracePlus wristbands is based on two different participant cohorts. Consequently, the observed differences cannot be attributed solely to the wearable devices, as cohort-specific and environmental factors may also have influenced the results. However, the behavioural and subjective data evaluations showed comparable trends across both studies, indicating that the experimental tasks elicited similar responses in the two cohorts. The exploratory nature of the comparison between the Empatica E4 and the EmbracePlus should therefore be considered when interpreting the results.

Furthermore, the controlled study environments and the study tasks do not fully reflect real-world conditions. Nevertheless, the tasks were selected carefully to simulate realistic physiological states such as cognitively demanding tasks, rest, physical activity and being startled. This approach enables controlled comparisons of wearable devices while maintaining some ecological relevance. Prior work has demonstrated the feasibility of using wearables such as the Empatica E4 in real-world work environments based on participants’ subjective experiences [50]. Further studies are needed to validate signal quality and suitability of research- and consumer-grade wearables for real-world empirical studies. In addition, future studies comparing wearable devices with clinical gold-standard reference systems would enable the assessment of signal accuracy alongside the relative device comparisons presented in this work. Replication studies with larger sample sizes would increase the statistical power to detect subtle physiological effects, such as those associated with different levels of cognitive load during the n-back task.

This study included the Pixel Watch 2 as a representative for consumer-grade wearables due to its integration of an EDA sensor, which comparable smartwatches lack. Only whilst trying to retrieve data from the watch did we notice that most data, including the EDA data, was uninterpretable and therefore unsuitable for data analysis. Consequently, the evaluation of the Pixel Watch 2 in this study was limited to processed heart rate and skin temperature measurements at no fixed sampling rate. In research settings, understanding the underlying preprocessing steps of physiological data is vital for reproducibility, which is why we cannot recommend the Pixel Watch 2 in its current configuration for empirical studies. However, other consumer-grade devices may offer better accessibility to raw data and should be validated in future studies. As new research- and consumer-grade wearables continue to emerge, it is essential to evaluate whether they offer improved accuracy and provide researchers with sufficient data access for meaningful physiological data analysis.

## 8. Conclusions

This work evaluated the comparability and reproducibility of physiological measurements from research- and consumer-grade wearables in controlled task- and event-based settings. Behavioural and subjective results confirmed that the study design reliably elicited distinct cognitive load levels and an event-based startle response, providing a valid basis for evaluating physiological responses. The results showed that wearable devices reliably capture large, relative physiological changes across conditions (i.e., sitting vs. physical activity) but are less reliable at capturing more subtle physiological responses, such as differences in cognitive load levels. Wearables differ in absolute values and their robustness to motion.

For empirical research, our findings suggest that the Pixel Watch 2 may be suitable for studies focusing on relative trends based on processed heart rate and skin temperature, especially in dynamic settings where robustness to motion is required. However, research-grade devices should be preferred when access to raw data, transparent preprocessing, and advanced metrics such as HRV or detailed EDA features are required. Across modalities, electrodermal activity provides more consistent task differentiation than PPG-derived measures under motion, while skin temperature captures slower, long-term changes.

The exploratory comparison between the Empatica E4 and EmbracePlus suggests that the newer EmbracePlus can achieve comparable overall performance, while indicating potential differences in sensitivity to specific signal characteristics, such as phasic EDA and short-term heart rate dynamics.

Wrist-worn devices were perceived as more comfortable and unobtrusive than finger-based sensors, highlighting the importance of device design for real-world applicability. Overall, careful device selection should be guided by the research question and aim, required signal processing transparency, and expected movement conditions.

## Figures and Tables

**Table 1:** Physiological parameters considered for analysis across the investigated wearables.

Physiological Parameter	Shimmer3 GSR+	Empatica E4	EmbracePlus	Pixel Watch 2
HR	✓	✓	✓	✓
HRV (RMSSD, pNN50)	✓	✓	✓	✗ *
EDA (SCL, cleanEDA, countSCRs)	✓	✓	✓	✗ *
TEMP (meanTEMP, rangeTEMP)	✗ **	✓	✓	✓

* Pixel Watch 2 records PPG and EDA data, but the exported data were not interpretable and were therefore not included in the analysis. ** The Shimmer3 GSR+ contains an internal device temperature sensor but does not provide a dedicated skin-temperature measurement; therefore, the data were excluded from the skin-temperature comparisons.

**Table 2:** Average percentage of data removed due to motion artefacts or value outliers. Removed outlier values may include interpolated peaks.

	Average Percentage of Data Removed Per Device			
Signal	Shimmer3 GSR+	Empatica E4	EmbracePlus	Pixel Watch 2
***Data removal due to motion artefacts (or inaccuracy)***				
PPG/HR	4.8%	6.1%	5.4%	6.5%
EDA	4.8%	3.7%	8.8%	-
TEMP	-	3.7%	6.1%	0%
***Data removal due to HR outliers (outside of 40–200 bpm; >40% change) from motion artefact cleaned signal***				
PPG/HR	3.9%	14.1%	7.8%	0%
***Data removal due to TEMP out of range (outside of 25–40 °C)***				
TEMP	-	0%	0%	0%

**Table 3:** Pre-processing pipeline comparison based on linear mixed-effects model analyses of the metric lnRMSSD on a dataset with a common subset of tasks across all pipelines.

Pre-Processing Pipeline	Motion Artefact Removal	IBI/HR Outlier Removal	N_tasks_	AIC	Marginal R^2^
P_minimal_	✗	✗	228	577.0	0.203
P_motion_	✓	✗	228	476.9	0.402
P_motion+HR_	✓	✓	228	554.1	0.442

**Table 4:** Analysis of the interaction effect: the full model was compared to the null model without the interaction effect for each physiological measure.

Measure	AIC_full model_	AIC_null_model_	χ^2^	df	*p*
lnHR	−379.94	−261.36	138.58	10	<0.001
lnRMSSD	512.44	513.72	11.276	5	0.0462
pNN50	1990.5	1995.9	15.44	5	0.0086
lnSCL	531.2	524.5	3.2936	5	0.6548
lnEDA	534.00	528.12	4.1217	5	0.532
countSCR	1707.8	1702.0	4.1252	5	0.5315
meanTEMP	475.27	489.26	23.986	5	<0.001
rangeTEMP	−487.98	−439.08	58.901	5	<0.001

**Table 5:** Analysis of the main effects wearable and task for the EDA measures.

Measure	Main Effect	AIC_full_model_	AIC_null_model_	χ^2^	df	*p*
lnSCL	wearable	531.2	664.16	144.95	6	<0.001
	task	531.2	553.1	41.901	10	<0.001
lnEDA	wearable	534.0	667.4	145.4	6	<0.001
	task	534.0	557.11	43.111	10	<0.001
countSCR	wearable	1707.8	1722.1	26.256	6	<0.001
	task	1707.9	1716.2	28.431	10	0.0015

**Table 6:** The interaction and main effects on the cardiac and EDA measures to analyze the startle event.

Measure	Effect	AIC_full_model_	AIC_null_model_	χ^2^	df	*p*
lnHR	wearable	−308.32	−292.91	27.408	6	<0.001
	startle phase	−308.32	−298.33	21.987	6	0.0012
	interaction	−308.32	−310.86	5.4539	4	0.2438
lnRMSSD	wearable	219.68	273.35	59.67	3	<0.001
	startle phase	219.68	234.44	22.761	4	<0.001
	interaction	219.68	216.02	0.3396	2	0.8438
pNN50	wearable	993.59	1041.61	54.013	3	<0.001
	startle phase	993.59	1000.79	15.199	4	0.0043
	interaction	993.59	990.32	0.7269	2	0.6953
lnSCL	wearable	281.39	355.41	80.024	3	<0.001
	startle phase	281.39	271.19	0	4	1
	interaction	281.39	274.49	0	2	1
lnEDA	wearable	281.39	358.75	83.359	3	<0.001
	startle phase	281.39	275.88	2.4879	4	0.6468
	interaction	281.39	277.57	0.1789	2	0.9144
countSCR	wearable	430.86	459.29	34.425	3	<0.001
	startle phase	430.86	440.48	17.614	4	0.0015
	interaction	430.86	430.39	3.5223	2	0.1718

**Table 7:** The interaction and main effects on the cardiac, electrodermal and skin temperature metrics to analyze the difference between the Empatica E4 and EmbracePlus devices during n-back and relaxation tasks.

Measure	Effect	AIC_full_model_	AIC_null_model_	χ^2^	df	*p*
lnHR	wearable	−139.17	−143.94	3.2241	4	0.5211
	task	−139.17	−138.17	13.004	6	0.043
	interaction	−139.17	−144.53	0.6399	3	0.8872
lnRMSSD	wearable	310.49	305.59	3.0978	4	0.5416
	task	310.49	304.62	6.1266	6	0.4092
	interaction	310.49	305.45	0.9605	3	0.8108
pNN50	wearable	1149.0	1142.7	1.7633	4	0.7792
	task	1149.0	1140.1	3.158	6	0.7888
	interaction	1149.0	1143.8	0.7942	3	0.8509
lnSCL	wearable	219.59	224.86	13.271	4	0.0100
	task	219.59	219.59	27.777	6	<0.001
	interaction	219.59	214.74	1.1469	3	0.7658
lnEDA	wearable	216.72	221.91	13.192	4	0.0104
	task	216.72	229.48	24.761	6	<0.001
	interaction	216.72	211.81	1.0921	3	0.779
countSCR	wearable	1119.8	1128.8	17.037	4	0.0019
	task	1119.8	1109.7	1.9126	6	0.09276
	interaction	1119.8	1115.0	1.1373	3	0.7681
meanTemp	wearable	33.476	30.568	5.0922	4	0.278
	task	33.476	24.878	3.402	6	0.757
	interaction	33.476	28.609	1.1331	3	0.7691
rangeTEMP	wearable	−393.65	−396.88	4.7707	4	0.3116
	task	−393.65	−389.12	16.53	6	0.0112
	interaction	−393.65	−394.99	4.6609	3	0.1984

**Table 8:** The interaction and main effects on the cardiac, electrodermal and skin temperature metrics to analyze the difference between the Empatica E4 and EmbracePlus devices during the startle event.

Measure	Effect	AIC_full_model_	AIC_null_model_	χ^2^	df	*p*
lnHR	interaction	−91.386	−88.985	6.4007	2	0.0408
lnRMSSD	wearable	228.96	227.01	4.0503	3	0.2561
	startle phase	228.96	234.88	13.925	4	0.0075
	interaction	228.96	228.07	3.1119	2	0.211
pNN50	interaction	897.19	905.61	12.418	2	0.00201
lnSCL	wearable	76.565	82.225	11.66	3	0.0086
	startle phase	76.565	82.362	13.797	4	0.008
	interaction	76.565	72.576	0.0130	2	0.9949
lnEDA	wearable	109.88	117.16	13.283	3	0.0041
	startle phase	109.88	116.79	14.912	4	0.0049
	interaction	109.88	107.44	1.5661	2	0.457
countSCR	interaction	606.26	609.01	6.7463	2	0.0343
meanTemp	wearable	−117.62	−119.27	4.3418	3	0.2268
	startle phase	−117.62	−121.42	4.1963	4	0.3801
	interaction	−117.62	−121.26	0.3598	2	0.8353
rangeTEMP	wearable	−551.98	−550.95	7.029	3	0.071
	startle phase	−551.98	−555.71	4.2702	4	0.3707
	interaction	−551.98	−552.65	3.3318	2	0.189

## Data Availability

Participant data is not made publicly available due to data privacy restrictions.

## Abbreviations

The following abbreviations are used in this manuscript:

PPG	Photoplethysmography
EDA	Electrodermal activity
TEMP	Skin temperature
EOG	Electrooculographic
ECG	Electrocardiogram
HR	Heart rate
HRV	Heart rate variability
IBI	Interbeat interval
RMS	Root mean square
ANOVA	Analysis of variance
SCL	Skin conductance level
SCR	Skin conductance response
RMSSD	Root mean square of successive differences
